# Improved power by collapsing rare and common variants based on a data-adaptive forward selection strategy

**DOI:** 10.1186/1753-6561-5-S9-S114

**Published:** 2011-11-29

**Authors:** Yilin Dai, Ling Guo, Jianping Dong, Renfang Jiang

**Affiliations:** 1 Department of Mathematical Sciences, Michigan Technological University, 1400 Townsend Drive, Houghton, MI 49931, USA

## Abstract

Genome-wide association studies have been used successfully to detect associations between common genetic variants and complex diseases, but common single-nucleotide polymorphisms (SNPs) detected by these studies explain only 5–10% of disease heritability. Alternatively, the common disease/rare variants hypothesis suggests that complex diseases are often caused by multiple rare variants with moderate to high effects. Under this hypothesis, the analysis of the cumulative effect of rare variants may thus help us discover the missing genetic variations. Collapsing all rare variants across a functional region is currently a popular method to find rare variants that may have a causal effect on certain diseases. However, the power of tests based on collapsing methods is often impaired by misclassification of functional variants. We develop a data-adaptive forward selection procedure that selectively chooses only variants that improve the association signal between functional regions and the disease risk. We apply our strategy to the Genetic Analysis Workshop 17 unrelated individuals data with quantitative traits. The type I error rate and the power of different collapsing functions are evaluated. The substantially higher power of the proposed strategy was demonstrated. The new method provides a useful strategy for the association study of sequencing data by taking advantage of the selection of rare variants.

## Background

Genome-wide association studies (GWAS) have been used successfully to detect associations between common genetic variants and complex diseases. However, common single-nucleotide polymorphisms (SNPs) detected by current GWAS explain only at most 5–10% of disease heritability [1]. One possible reason could be that another type of variant, rare variants, has not been considered in the current GWAS. Recent studies have shown that common diseases can be caused by functional variants with a wide spectrum of allele frequencies, including rare alleles [2-4]. GWAS on common SNPs are based on the currently popular common disease/common variants hypothesis for complex disease etiology; these studies are well suited for detecting genetic variants with high allele frequencies and relatively small to modest genetic effects. There are some difficulties in identifying variants based on the alternative common disease/rare variants hypothesis, which suggests that complex traits are caused collectively by multiple rare variants with moderate to high effects. Under this hypothesis, the analysis of the cumulative effect of rare variants may become crucial for discovering the missing genetic variation from traditional GWAS.

With the development of next-generation sequencing technologies, more rare variants can be genotyped, so the analysis of associations between rare variants and diseases becomes possible. It is well known that traditional GWAS lack power for detecting rare variants. More powerful tests are needed to analyze resequencing genetic data. Recently, Li and Leal [5] proposed a strategy that collapses all the rare variants across a functional region. The idea behind this strategy is to assume that each rare variant in a functional region contributes to a disease, and collapsing genotypes across variants will result in enriched association signals.

Several tests based on different collapsing strategies for case-control studies have been proposed. One is the cohort allelic sum test (CAST) [6], in which the number of individuals with one or more mutations in a group (e.g., a gene) are compared between case subjects and control subjects. Because CAST deals only with rare variants, the combined multivariate collapsing (CMC) method [5] generalized it by performing a multivariate test with common variants and collapsing scores of rare variants. A weighted-sum statistic [7] is another method that collapses both common and rare variants by adding different weights based on allele frequencies, assuming that rare variants have a high effect compared with common variants. With the regression approaches proposed by Morris and Zeggini [8], these methods can also be extended to quantitative phenotypes. In addition, the power of a single-marker test is usually low because of the lack of genetic variant information and the need to adjust for multiple corrections. Multiple-marker tests might also lose power as a result of higher degrees of freedom. The collapsing methods can avoid drawbacks from both single-marker tests and multiple-marker tests by considering all the genetic variant information with only 1 degree of freedom.

However, the collapsing methods may not be robust and could be seriously impaired by misclassification of collapsing regions [5]. Regions can usually be defined by genes, SNP allele frequencies, or variant functionality. If all rare variants within a collapsing region have the same positive or negative effect on a disease, then the association signal could be amplified; however, if collapsing combines functional and nonfunctional variants, this would adversely affect power. To address this problem, we develop a data-driven forward selection strategy in which a common variant is first chosen as a base to collapse a specific region and then rare variants are selected to be collapsed with the base SNP. The proposed method is well suited for detecting regions in which common and rare variants have the same genetic effect on a disease, especially when the association signal of common variants cannot be identified by traditional GWAS. The new method is robust to the size of the region and can efficiently deal with noise caused by misclassification of noncausal rare variants. We apply our method to the Genetic Analysis Workshop 17 (GAW17) unrelated individuals data with quantitative traits Q1 and Q2. The proposed method works for quantitative traits.

## Methods

### Data preprocessing

We analyze the GAW17 unrelated individuals data set, with 200 replicates of simulated phenotypes Q1 and Q2, to compare the power of different tests. There are 697 samples consisting of 209 case subjects and 488 control subjects. The data set contains 24,487 SNPs within 3,205 genes generated using real sequence data from the 1000 Genomes Project. We define rare variants as SNPs with a minor allele frequency less than 0.01 and perform collapsing within each gene. Because collapsing methods do not work well if the region includes too few SNPs, we first filter the genes according to a criterion of having at least 10 variants with one or more common SNPs. After filtering, we have 553 genes for analysis. The analysis is performed with the knowledge of the underlying simulating model.

To control the false-positive rate, we adjust phenotypes for the effects of confounding variables and population stratification. We perform a linear regression of the phenotypes from the first replicate on the variables Sex, Age, and Smoking status to select confounding variables. The results are shown in Table 1. Variables with a *p*-value greater than 0.05 are selected as covariates for the adjustment. The top five eigenvectors from Eigenstrat [9,10] are also considered covariates. For each replicate, residuals of the multivariate linear regression of phenotypes on all the selected covariates are regarded as the adjusted phenotypes.

**Table 1:** *p*-Value for the selection of covariates for Q1 and Q2

Covariate	*p*-value for Q1	*p*-value for Q2
Intercept	1.19 × 10^−9^	0.149
Age	<2 × 10^−16^	0.353
Sex	0.564	0.315
Smoke	1.09 × 10^−12^	0.329

Age and Smoke are selected as the covariates to adjust the phenotype Q1; no covariates were used for the adjustment of Q2.

### Data-adaptive forward selection

In brief, our strategy is as follows. We start with the most significant common SNP within one region. Rare variants are then added to the collapsed set one at a time until there are no variants remaining or until there is no visible improvement in the goodness-of-fit of the fitted model. More specifically, assume that there are *m* common variants and *n* rare variants within a certain predefined genomic region. Let *x* denote the genotype for all samples of a certain common variant, and let *g* denote the rare variant. Our strategy consists of the following steps.

**Step 1.** Build a linear model on each common SNP. The SNP with the largest genetic effect as measured by the *F* statistic of the linear regression is selected as the base of the collapsing function for this region:(1)

*F* is calculated based on:(2)

**Step 2.** Collapse each rare variant with the base SNP in this region according to a specific collapsing function. Perform a linear regression on each collapsed score Collapse(*S*, *g_i_*). Based on the *F* statistics, variants with the most significant values are then selected as the base for the next procedure:(3)

*F*_new_ is calculated based on:(4)

**Step 3.** If *F*_new_ >*F*, then update *S*, *F*, and *n* with *S*_new_, *F*_new_, and *n* − 1. Repeat step 2 until either *F* no longer increases or *n* = 0.

When the selection procedure is finished, the test statistic FS is defined as:(5)

which is the absolute value of the *t* statistics in the linear regression model:(6)

where *S*_final_ is the final collapsed score after the selection. Under the null hypothesis, the selection procedure drives the statistics in two different directions; taking the absolute value allows us to proceed with the normalization step, described in the next subsection.

### Genome-wide permutation test

To correct the bias resulting from selection and obtain the global empirical *p*-value, we perform a genome-wide permutation test. Assume that *M* permutations are performed for *k* candidate genes. Let FS*_i_* be the observed *t* statistics for the *i*th gene, and let  be the observed *t* statistics for the *i*th gene on the *j*th permutation. To compare the genetic effect across genes, we normalize the statistics using the estimated mean and variance from the permutation and obtain the adjusted statistic FS_adj_*_i_*:(7)

and:(8)

where:(9)

and:(10)

Let:(11)

be the maximum value of the observed statistics in the *j*th permutation. The global *p*-value of the *i*th gene is the proportion of *m_j_* > FS_adj_*_i_*:(12)

### Comparison of tests

We investigate the performance of our forward selection strategy with three collapsing methods: (1) the indicator method, (2) the sum method, and (3) the weighted-sum method. Let *r_ij_* represent the genotype for the *i*th individual at the *j*th locus. The collapsing functions are then defined as follows. The indicator function:(13)

is a function of the presence or absence of any minor allele in any region within an individual, which was first used in CAST [6]. The sum function:(14)

is a function that describes the overall effect at any region within an individual. It has the same effect as proportion coding. Both the indicator and sum functions have been demonstrated to be powerful in the detection of associated rare variants [8]. The weighted-sum function is:(15)

where the weight is calculated by:(16)

(there is one unreadable character of the right side of (16) in my computer. Please make sure it is same as before. It is q hat)

and:(17)

and *n* is the total number of individuals.

We estimate allele frequencies by jointly considering all subjects, because we could not follow the suggestion to estimate allele frequencies from unaffected individuals [7] when dealing with quantitative traits.

The test statistics of the indicator, sum, and weighted-sum functions without a forward selection strategy are denoted *T*_ind_, *T*_sum_, and *T*_ws_, respectively, and their *p*-values can be calculated from the *t* distribution and adjusted by the Bonferroni correction. Statistics *T*_ind_ and *T*_sum_ deal only with the rare variants, whereas *T*_ws_ considers both common and rare variants.

The corresponding statistics obtained with our forward selection strategy are denoted FS_ind_, FS_sum_, and FS_ws_. Another test statistic, denoted *T*_com_, is also obtained by using linear regression on the most significant common variant in a region. A comparison of the proposed strategy with *T*_com_ allows us to access the effect of adding rare variant information to common variant information in a specific genetic region.

## Results

### Type I error and power

We analyzed 553 filtered genes on the 200 replicates for phenotypes Q1 and Q2. The type I error rate of the test and the power of the test are defined as follows. Take Q1 as an example; there are 4 functional and 549 nonfunctional genes. At a given significance level *α*, if the adjusted or global *p*-value for a gene is greater than *α*, we would reject the null hypothesis. Next, we consider (number of tests that rejected the null hypothesis)/[200(549)] for the 549 nonfunctional genes as the type I error rate of the test. Because different tests should adapt to different types of genes, power is calculated by (number of tests rejected at the null hypothesis)/200 for each functional gene.

We found that the type I error of tests using the weighted-sum function was inflated for Q1. To have a fair comparison, we selected a different significance level for each test to have the type I error rates of all tests at the same level. Some permutation tests cannot have exact 5% type I error because there were only 1,000 permutations. Therefore we chose a significance level so that all tests had a well-controlled false-positive rate of about 6% for Q1 and 5% for Q2 (Table 2). The power of the tests was calculated based on the same significance levels as the type I error. Table 3 lists all the genes with power greater than 5% according to at least one test.

**Table 2:** Significance level (*α*) and type I error rate for Q1 and Q2

Trait	*T*_ind_	*T*_sum_	*T*_ws_	FS_ind_	FS_sum_	FS_ws_	*T*_com_
Q1: *α*	0.042	0.038	0.0171	0.052	0.035	0.003	0.038
Q1: type I	6%	6%	6%	5.5%	6%	6%	6%
Q2: *α*	0.041	0.03	0.103	0.06	0.054	0.083	0.053
Q2: type I	5%	5%	5%	5%	5%	5%	5%

*T*_ind_, *T*_sum_, and *T*_ws_ use adjusted *p*-values by the Bonferroni correction; FS_ind_, FS_sum_, FS_ws_, and *T*_com_ use the global permutation *p*-value. The power of tests is calculated on the basis of the same significance levels as the type I error.

**Table 3:** Power of seven tests for Q1 and Q2

Phenotype	Gene	*T*_ind_ (%)	*T*_sum_ (%)	*T*_ws_ (%)	FS_ind_ (%)	FS_sum_ (%)	FS_ws_ (%)	*T*_com_ (%)
Q1	*FTL1*	5	9.5	80.5	95	99.5	71	100
	*KDR*	2	4	21	9.5	15.5	28	1.5
Q2	*SREBF1*	5	6	2	0	0	0.5	0
	*SIRT1*	1	2.5	7.5	0	0.5	8	0
	*VNN3*	0	0	1	6	5.5	4	2.5

*T*_ind_, *T*_sum_, and *T*_ws_ are tests of collapsing-function-based linear regression without the forward selection procedure, and FS_ind_, FS_sum_, and FS_ws_ are forward-selection-based tests. Collapsing functions include the indicator, sum, and weighted-sum functions. The power of tests is calculated on the basis of the same significance levels as the type I error.

For Q1, *FTL1* was detected by *T*_com_ with 100% power, which indicated that the common SNP had a strong effect on the disease. However, *T*_com_ is not optimal for evaluating the proposed strategy. Gene *KDR* is the case we want to consider, because *T*_com_ became underpowered, which indicated that the effects of common SNPs were not significant. For *KDR*, FS_ws_ achieved the highest power, followed by *T*_ws_, FS_sum_, and FS_ind_. We also found that tests considering both common and rare variants achieved higher power than those that considered only rare variants (*T*_ind_ and *T*_sum_) or common variants (*T*_com_). All forward-selection-based tests were ranked at the top for *KDR*, which demonstrates the potential power of the forward selection strategy. For Q2, all tests became underpowered. Three genes (*SREBF1*, *SIRT1*, and *VNN3*) were detected by at least one test with power greater than 5%. FS_ws_ achieved the highest power (8%) in detecting *SIRT1*.

## Discussion

We have proposed a data-adaptive forward selection strategy for genetic association studies with multiple common and rare variants. The proposed test is aimed at selecting rare variants for collapsing that best amplify the association signal between functional regions and phenotypes. Traditional collapsing methods do not have the option of selectively collapsing only functional rare variants with the same effects on the risk of disease; thus they may be underpowered by misclassification in collapsing regions. The major advantage of our method is that it can selectively collapse rare variants with the same genetic effect as the common variant in the functional region, thus leading to a more significant association signal. To correct the bias resulting from selection and to alleviate the computational burden, we performed a genome-wide permutation test. We evaluated the power of our method using different collapsing functions, based on the same type I error. The results show that the proposed method has substantially higher power across different collapsing strategies. The way to select rare variants for each functional region also suggests that our method may have higher power to detect functional regions with an either positive (damage) or negative (protective) effect on the disease traits, even if no common variant is associated with the disease, so long as enough rare variants collectively affect the disease. The forward selection strategy could also be a powerful tool by adding different weights based on allele frequencies in order to lower the effect of common variants.

## Conclusions

We developed a data-adaptive forward selection procedure by collapsing a common variant with selected rare variants. The validity and substantially higher power of the proposed strategy were demonstrated using the GAW17 data. The method provides a useful strategy for association studies of sequencing data by taking advantage of the selection of rare variants.

## Competing interests

The authors declare that they have no competing interests.

## Authors’ contributions

YD contributed in the development of the statistical test, provided data analysis strategies, and drafted the manuscript. LG carried out part of the programing work. RJ and JD both supervised the whole process and participated in drafting the manuscript. All authors read and approved the manuscript.
